# Terminology and Definitions of Racial Health Equity in Prominent Health Websites: Systematic Review

**DOI:** 10.2196/64868

**Published:** 2025-07-23

**Authors:** Mahederemariam Bayleyegn Dagne, Elizabeth A Terhune, Miriam Barsoum, Ana Beatriz Pizarro, Anita Rizvi, Damian K Francis, Meera Viswanathan, Nila A Sathe, Vivian Welch, Tiffany Duque, Robert W Turner II, Tamara A Baker, Patricia C Heyn

**Affiliations:** 1 Center for Optimal Aging, Marymount University, Arlington, VA, United States; 2 Office of Research, Marymount University, 2807 N Glebe Road 26, Arlington, VA, 22207, United States, 1 7032843810; 3 Department of Orthopedics, University of Colorado, Anschutz Medical Campus, CO, United States; 4 Clinical Research Center, Fundación Valle del Lili, Cali, Colombia; 5 Cochrane Collaboration, Central Executive Team, London, United Kingdom; 6 School of Psychology, University of Ottawa, Ottawa, ON, Canada; 7 School of Health and Human Performance, Georgia College and State University, Milledgeville, GA, United States; 8 RTI International-University of North Carolina Evidence-Based Practice Center US Cochrane Affiliate, Research Triangle Park, NC, United States; 9 Bruyere Health Research Institute, Ottawa, ON, Canada; 10 School of Epidemiology and Public Health, University of Ottawa, Ottawa, ON, Canada; 11 Department of Population Health Sciences, Duke University School of Medicine, Durham, NC, United States; 12 Department of Psychiatry, The University of North Carolina, Chapel Hill, NC, United States; 13 Strauss Health Sciences Library, University of Colorado, Anschutz Medical Campus, Aurora, CO, United States

**Keywords:** health equity, race, web-based health information, definitions, websites, thematic analysis, sentiment analysis, racial health equity, health care, systematic Review, public health, health information, promotion, inclusive, racial, United States

## Abstract

**Background:**

The websites of prominent public health and health care organizations play pivotal roles in ensuring access to quality health information, including information guiding health equity. Several initiatives have been developed in the United States to promote equitable, fair, and inclusive health information and practices across prominent health websites. Currently, health disparities across racial groups are recognized as a critical public health problem. Simultaneously, the use of the term “racial health equity/equities” has been rising in academic literature. However, the definition and findability of “racial health equity/equities” information have not yet been evaluated in health websites. Thus, we used a systematic review approach to assess the findability and availability of racial health equity terminology and definitions across prominent health organization websites.

**Objective:**

The objective of this study was to systematically evaluate the definitions and findability of “racial health equity/equities and related terms” on prominent health organizations’ websites.

**Methods:**

We conducted a systematic review of websites from government agencies, professional organizations, and selected health care organizations with relevance to the US health care system. Google and the US Digital Analytics program were used for initial searches. Definitions, terms, and accompanying citations for racial health equity terms, including “racial health inequity” or “racial health disparities,” were extracted from all websites. A findability tool was developed to evaluate the ease of finding the terms and definitions, with ratings ranging from “very easy” to “very difficult.” Additionally, we analyzed the themes and sentiments of the retrieved definitions.

**Results:**

We analyzed 69 websites from prominent health organizations. Approximately half (n=31) of the websites lacked any definitions for racial health equity and related terms, and of the 38 that included definitions, most did not include citations. The definitions varied across websites, and most were rated as “very difficult” to find.

**Conclusions:**

This study highlights the absence of a systematic, standardized, and accurate approach to organizing, defining, and presenting racial health equity information on prominent health websites. Specifically, there is a lack of consistent definitions for racial health equity and related terms across prominent health organization websites.

## Introduction

Significant disparities in health care access [1,2], health outcomes [2], and health literacy [3,4] exist across racial and ethnic groups within the United States. Although racial health disparities have been discussed for decades, there has been renewed attention for improving the health of underserved populations in recent years, particularly in the wake of racial justice protests [5] and the COVID-19 pandemic, which disproportionately affected Black, Hispanic or Latino, and Indigenous populations [6]. The recent call for advancing racial equity by the White House [7,8] has increased initiatives addressing inequities.

Subsequently, there has been a significant rise in the use of “health equity/equities” terminology across health websites [9] and academic literature [10,11]. Public health and health care organizations, such as the Centers for Disease Control and Prevention and the American Public Health Association, use their websites to disseminate crucial health information [12], including guidance on health equity practices [7,8]. As most adults in the United States report that web-based searches are their first sources of health information [13], the ability to easily find health information on the web is critical for patients and their caretakers [14].

Ambiguous health equity definitions can create variability in understanding across disciplines, demographics, and contexts, leading to confusion and misinterpretation [15,16]. This ambiguity presents a challenge in formulating, monitoring, and evaluating equitable health policies and practices, particularly those addressing racial disparities. Therefore, establishing clear definitions on websites serves multiple crucial functions, including fostering shared comprehension, avoiding ambiguity, and facilitating the assessment of intervention efficacy [17].

Despite comprehensive reporting guidelines for health research in peer-reviewed journals [18,19], no current widely accepted guidelines have been developed for websites of health institutions [20,21]. Although the American Medical Association developed guidelines for websites that provided medical and health information in the early 2000s [21], most organizations have their own standards and practices for displaying health information, including website design. Additionally, while there are tools for consumers to evaluate the accuracy and legitimacy of information presented on health websites, most do not address findability of the information [22,23]. Furthermore, most tools are designed to guide the end users on how to evaluate websites [24,25], placing a higher burden on the website user. This lack of comprehensive guidance results in significant variability in the reliability and accuracy of web-based health information, making it challenging for the public to identify credible sources [26,27]. Therefore, it is essential that prominent health websites provide clear and easily findable information [28-30].

Thus, we use a systematic review approach to understand how prominent US and worldwide health organizations’ websites adopt and display definitions and terms informing the public about racial health equity. For this study, “prominent health organizations” *are defined as reputable public health and health care organizations that are known to provide evidence-based health information to the public, have high website traffic, and promote public health as part of their mission*.

The key questions (KQs) for this study are as follows:

KQ1: How easy is it to find definitions of racial health equity/equities and related terms on prominent health organization websites?

KQ2: How are racial health equity/equities and related terms included and defined on prominent health organization websites?

KQ3: What common definitions, terms, themes, sentiments, and citations related to racial health equity/equities and related terms will emerge from the identified health organization websites?

## Methods

We used a systematic review approach in accordance with guidelines established by the PRISMA (Preferred Reporting Items for Systematic Reviews and Meta-Analyses) statement (Checklist 1) to evaluate how prominent health organizations display, define, and use health equity and racial health equity terminology within their websites [31,32].

### Protocol Registration

This study was part of a project titled “Centering Racial Health Equity in Evidence Syntheses” funded by the Robert Wood Johnson Foundation [10,33], intending to evaluate and provide recommendations for centering racial health equity within evidence syntheses [34]. The protocol was reviewed and guided by an external steering committee composed of interest holders and is available through Open Science Framework and was published [35].

### Inclusion and Exclusion Criteria

The scope of this study was to systematically evaluate how racial health equity and related terms are defined and displayed on prominent health organizations’ websites [12,36]. US-specific and global health websites (eg, World Health Organization) were included, which included websites from federal health organizations, nonprofit organizations, private foundations, associations, and professional societies. Included websites had to include public health as part of their mission, as determined from the “About” page. Websites that focused exclusively on 1 state or a particular geographic area were excluded (eg, state health organizations, for-profit health organizations, universities, and hospitals). (Table S1 in Multimedia Appendix 1).

### Website Search

A Google search was first used to identify a list of websites for review using the search terms “public health organizations” and “health organizations in the US” from March until May 2023. The 51 organizations listed on Google were assessed and screened according to our inclusion criteria. Additionally, we screened the top 20 US government health websites with the highest traffic (number of monthly visitors to a website) according to the US Government Google Analytics Digital Analytics Program [37] (Figure S1 in Multimedia Appendix 1 [37]). We also reviewed the 27 National Institutes of Health and the 16 Health and Human Services organizations. Finally, we reviewed 7 additional relevant health organizations’ websites based on experts' recommendations. A total of 121 websites were included for systematic synthesis.

### Website Selection

A website list was generated and 2 members of the study team (MBD plus ET or MB) reviewed the website home page to determine final inclusion. Disagreements were resolved by discussion with a third reviewer (PH).

### Search Terms for Definitions

A comprehensive list of terms representing racial health equity concepts was compiled for analysis. The terms included health equity/equities, health inequity/inequities, health disparity/disparities, racial health equity/equities, racial health inequity/inequities, and racial health disparity/disparities.

### Data Extraction

The data extracted included website demographics (website URL, country, and organization type), inclusion of key terms, and word-for-word definitions of racial health equity terms and concepts, including citations used for the definitions. An Excel spreadsheet was used to store the data from all included websites. The data extraction process and tool were first tested for congruity between 2 independent (MBD and ET or MB) reviewers. A second reviewer verified all extracted (MB) data.

### Racial Health Equity and Related Terms’ Definitions Findability Assessment

**Figure 1.**
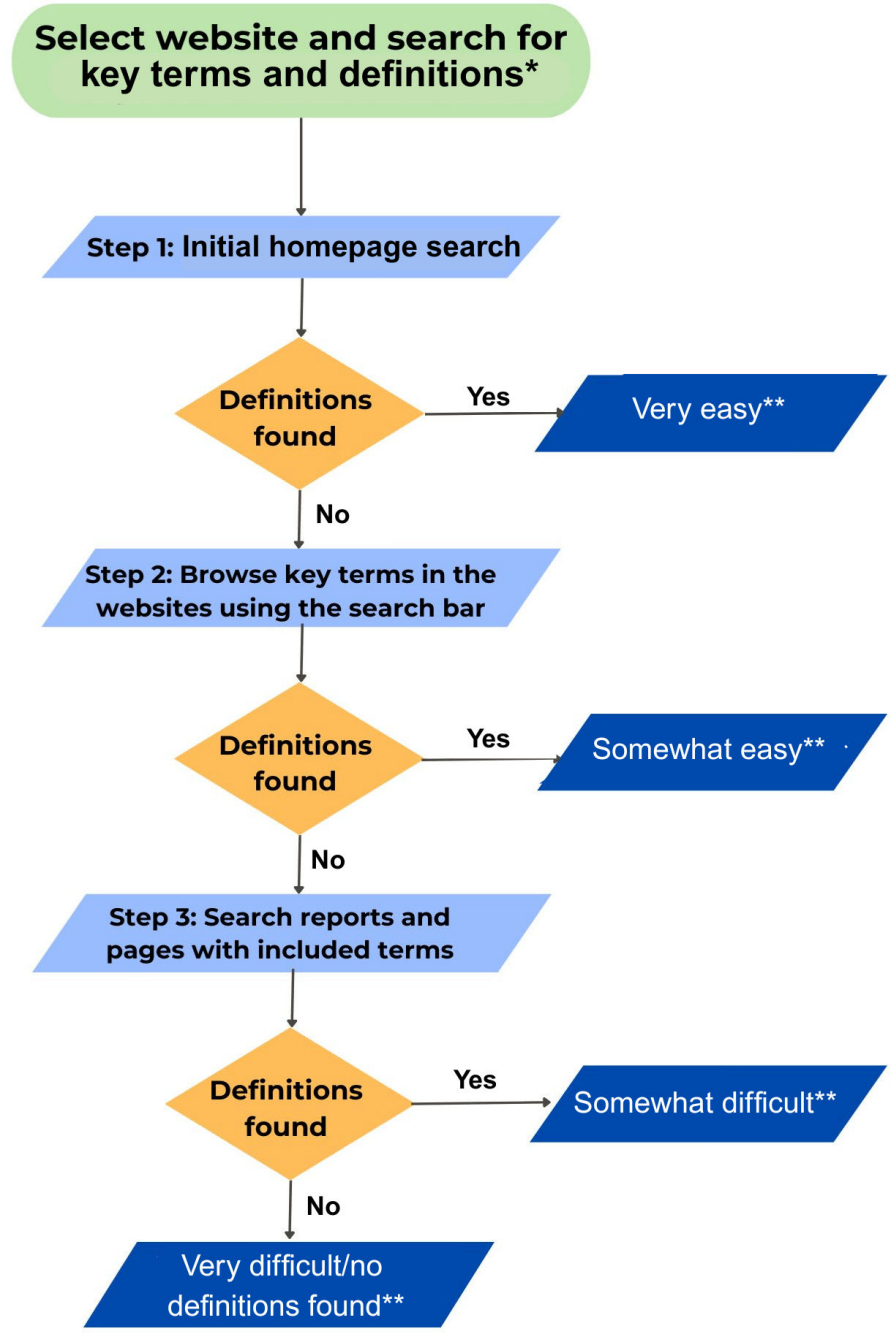
Procedure for browsing websites to find definitions and assigning findability. *Terms for extraction (racial health equity, racial health inequity, racial health disparity, health equity, health inequity, and health disparity). **Assigned findability score (0=Very difficult/no definitions found, 1=Somewhat difficult, 2=Somewhat easy, and 3=Very easy).

Findability within a website is determined by the user ability to find specific information easily, which enhances the user experience and increases interaction with the website [38-40]. As there were no existing website findability tools applicable to our research questions, we developed a project-specific findability tool for definitions. The findability tool was used to classify the ease of finding definitions for all terms identified for extraction in each website using a Likert scale [41] of “very difficult/no definitions found,” “somewhat difficult,” “somewhat easy,” or “very easy,” through a stepwise approach (Figure 1).

In the stepwise approach, we assigned a score from 0 (very difficult/no definitions found) to 3 (easy) for each reviewed term on all websites. If the definition for the reviewed term was found on the website home page, the findability for the definition was assigned as “very easy” with a score of 3 for the term. Following this initial review, a search for each term was performed by using the search bar and opening and reviewing the intra-site links of the resulting pages. If a definition was retrieved, findability was assigned as “somewhat easy” with a score of 2. Third, we reviewed any reports that included the terms from the previous search result. We then documented definitions. If a definition was located at this stage, the findability was designated as “somewhat difficult” with a score of 1. Finally, a findability designation of “very difficult /no definitions” with a score of 0 was assigned if no definitions were located following the completion of the stepwise search procedure.

For a systematic methodological approach to review websites, a 60 minutes maximum time was allocated for each website review process [42-44]. After compiling the terminological data from each website, we summed the individual definition term findability score to calculate an overall findability score for each site. A website where definitions were easily visible and accessible was classified as “very easy,” resulting in a maximum aggregate score of 18. On the other hand, a website where definitions were very difficult to locate, or entirely absent, received a score of 0, indicating the lowest level of findability. Therefore, if the sum of the individual terms’ findability score was from 13 to 18, the overall rating for the website was labeled as "very easy.” If the total score was from 7 to 12, it was considered “somewhat easy.” If the score ranged from 1 to 6, the findability was categorized as "somewhat difficult,” and a score of 0 was classified as “very difficult/no definitions found.” (Multimedia Appendix 2).

### Interrater Reliability

Two researchers (MBD and MB) performed the findability assessment for each website. The κ statistic [45] was used to analyze the interrater reliability of the findability scale for this study. Final ratings were determined by consensus.

### Thematic Analysis

Thematic analysis was conducted for included definitions to identify which prominent concepts were representing the definitions. The thematic analysis used the 2006 Braun and Clark framework [46,47]. The framework consists of 6 steps: familiarization with the definitions, initial coding, creation of themes, review of themes, definition of themes, and writing of final thematic analysis. Themes were developed separately by 2 members of the study team (MBD and MB), and any disagreements were resolved through discussion (Table S2 in Multimedia Appendix 1). The finalized themes were then reviewed by a third member of the team (ET).

### Sentiment Analysis

A sentiment analysis is a text analysis tool used to evaluate emotion and impartial tone in words indicated by polarity and subjectivity, respectively. Polarity is classified as positive, negative, or neutral. The scores range from −1 (completely negative) to 1 (completely positive) and a neutral range −0.05 to 0.05 [48]. Subjectivity is ranked by the level of impartiality found in the language ranging from 0 (objective) to 1 (subjective). The Vader and TextBlob Natural Language Processing tools were used for sentiment analysis. The Vader Natural Language Processing tool was used to measure polarity [49] and TextBlob was used for the subjectivity analysis [48-50].

## Results

### Summary of Search

**Figure 2.**
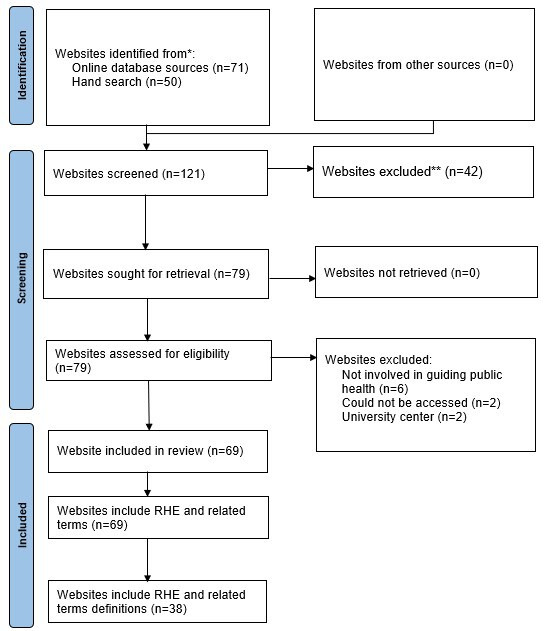
PRISMA (Preferred Reporting Items for Systematic Reviews and Meta-Analyses) diagram for included and excluded websites.*Gray literature using the search string: Public Health Organizations” (n=51), Health and Human Services (n=16), National Institutes of Health (n=26), and US Government Google Analytics Digital Analytics Program (n=20). **For-profit health organizations, medical university and colleges, and state health organizations. RHE: racial health equity.

The initial keyword search resulted in a list of 121 potential websites for inclusion. We excluded 41 websites after initial screening of the website home page for not meeting the inclusion criteria (see PRISMA diagram, Figure 2). Reasons for exclusion were non–US-based organizations (n=7), state health organizations (n=15), for-profit health organizations (n=10), universities (n=5), and hospitals (n=4). We excluded 10 additional websites after full screening of the website because the mission of the organizations was not within the scope of our study (n=6), could not be accessed (n=2), or was a university center (n=2). After full screening, 69 websites were included and the following terms were identified and included for analysis: “racial health equity/equities,” “racial health inequity/inequities,” “racial health disparities/disparities,” “health equity/equities,” “health disparity/disparities,” and “health inequity/inequities.”

### Characteristics of Included Websites

The representation of the websites included government health organizations (n=41), public (n=22), intergovernmental institutes (n=5), and private foundations (n=1). Organizations with primary or exclusive relevance to countries other than the United States were excluded. Of the 69 included websites, 62 were US-based while 7 addressed global health. The complete characteristic information for the included websites is shown in Table 1.

**Table 1.** Website characteristics and definitions findability score (accessed from March 3, 2023, to April 30, 2023).

Organization (access date)	Overall findability of terms and definitions^a^	Type of organization
Academy Health (April 30, 2023)	Somewhat difficult	Public
Administration for Children and Families (March 19, 2023)	Very difficult/no definitions found	Government
Administration for Community Living (March 22, 2023)	Very difficult/no definitions found	Government
Alzheimer’s Association (March 16, 2023)	Somewhat difficult	Public
American Academy of Nursing (April 30, 2023)	Very difficult/no definitions found	Public
American Academy of Pediatrics (May 3, 2023)	Somewhat difficult	Public
American Cancer Society (April 30, 2023)	Somewhat difficult	Public
American College of Physicians (April 30, 2023)	Somewhat difficult	Public
American Heart Association (March 23, 2023)	Somewhat difficult	Public
American Lung Association (March 23, 2023)	Very difficult/no definitions found	Public
American Medical Association (March 16, 2023)	Somewhat difficult	Public
American Public Health Association (March 9, 2023)	Somewhat difficult	Public
American Red Cross (March 23, 2023)	Very difficult/no definitions found	Public
Association Of Public Health Laboratories (March 16, 2023)	Very difficult/no definitions found	Public
Campbell Organization (April 30, 2023)	Somewhat difficult	Public
Centers for Disease Control and Prevention (March 12, 2023)	Somewhat difficult	Government
Centers for Medicare & Medicaid Services (March 12, 2023)	Somewhat difficult	Government
Cochrane (April 6, 2023)	Somewhat difficult	Intergovernmental
Fogarty International Center (April 5, 2023)	Very difficult/no definitions found	Government
Indian Health Services (March 23, 2023)	Very difficult/no definitions found	Government
National Academy of Medicine (Institute of Medicine) (April 5, 2023)	Somewhat difficult	Public
National Association of Community Health Centers (March 23, 2023)	Very difficult/no definitions found	Public
National Cancer Institute (April 2, 2023)	Somewhat difficult	Government
National Center for Advancing Translational Sciences (April 5, 2023)	Very difficult/no definitions found	Government
National Center for Complementary and Integrative Health (April 5, 2023)	Very difficult/no definitions found	Government
National Eye Institute (April 2, 2023)	Very difficult/no definitions found	Government
National Heart, Lung, and Blood Institute (April 2, 2023)	Very difficult/no definitions found	Government
National Human Genome Research Institute (April 2, 2023)	Very difficult/no definitions found	Government
National Institute of Allergy and Infectious Diseases (April 2, 2023)	Somewhat difficult	Government
National Institute of Arthritis and Musculoskeletal and Skin Diseases (April 2, 2023)	Very difficult/no definitions found	Government
National Institute of Biomedical Imaging and Bioengineering (April 2, 2023)	Very difficult/no definitions found	Government
National Institute of Child Health and Human Development (April 2, 2023)	Very difficult/no definitions found	Government
National Institute of Dental and Craniofacial Research (April 3, 2023)	Very difficult/no definitions found	Government
National Institute of Diabetes and Digestive and Kidney Diseases (April 3, 2023)	Somewhat difficult	Government
National Institute of Environmental Health Sciences (April 4, 2023)	Very difficult/no definitions found	Government
National Institute of General Medical Sciences (April 4, 2023)	Somewhat difficult	Government
National Institute of Mental Health (April 4, 2023)	Somewhat difficult	Government
National Institute of Neurological Disorders and Stroke (April 4, 2023)	Very difficult/no definitions found	Government
National Institute of Nursing Research (April 4, 2023)	Somewhat difficult	Government
National Institute on Aging (April 2, 2023)	Somewhat difficult	Government
National Institute on Alcohol Abuse and Alcoholism (April 2, 2023)	Very difficult/no definitions found	Government
National Institute on Deafness and Other Communication Disorders (April 2, 2023)	Very difficult/no definitions found	Government
National Institute on Drug Abuse (April 3, 2023)	Somewhat difficult	Government
National Institute on Minority Health and Health Disparities (April 4, 2023)	Somewhat difficult	Government
National Institutes of Health (March 12, 2023)	Somewhat difficult	Government
National Institutes of Health Clinical Center (April 5, 2023)	Very difficult/no definitions found	Government
Office for Human Research Protections (April 6, 2023)	Very difficult/no definitions found	Government
Office of Climate Change and Health Equity (April 6, 2023)	Somewhat difficult	Government
Office of Disease Prevention and Health Promotion (April 6, 2023)	Somewhat difficult	Government
Office of Minority Health (March 23, 2023)	Very difficult/no definitions found	Government
Office on Women’s Health (April 6, 2023)	Very difficult/no definitions found	Government
Patient-Centered Outcomes Research Institute (PCORI) (March 19, 2023)	Somewhat difficult	Public
Public Health Institute (March 12, 2023)	Very difficult/no definitions found	Public
Robert Wood Johnson Foundation (April 30, 2023)	Somewhat difficult	Public
Substance Abuse and Mental Health Services Administration (March 19, 2023)	Somewhat difficult	Government
The Agency for Healthcare Research and Quality (March 12, 2023)	Somewhat difficult	Government
The Carter Center (March 16, 2023)	Somewhat difficult	Public
The Commonwealth Fund (March 12, 2023)	Very difficult/no definitions found	Private
The Global Health Council (March 12, 2023)	Somewhat difficult	Public
The Health Resources and Services Administration (March 12, 2023)	Somewhat difficult	Government
The Pan American Health Organization (March 12, 2023)	Somewhat difficult	Intergovernmental
Trust for America’s Health (March 12, 2023)	Somewhat difficult	Public
US Preventive Services Task Force (March 19, 2023)	Very difficult/no definitions found	Public
US Public Health Service (March 23, 2023)	Very difficult/no definitions found	Government
United Nations (April 5, 2023)	Very difficult/no definitions found	Intergovernmental
United Nations Children’s Fund USA (April 6, 2023)	Very difficult/no definitions found	Intergovernmental
United States Department of Health and Human Services (March 12, 2023)	Somewhat difficult	Government
US Food and Drug Administration (March 16, 2023)	Very difficult/no definitions found	Government
World Health Organization (March 12, 2023)	Somewhat difficult	Intergovernmental

a Findability indicates the level of ease with which any definitions could be found using the findability tool developed for definitions (see the “Methods” section).

### Results of KQs

#### KQ1: How Easy Is It to Find Definitions of Racial Health Equity/Equities and Related Terms on Prominent Health Organization Websites?

To determine the degree of findability for definitions of racial health equity and related terms within each website, we developed a project-specific findability tool (see the “Methods” section). The score for racial health equity/equities and racial health inequity/inequities was “very difficult/no definitions found” across all 69 websites. Alternatively, for racial health disparities, 1.4% (n=1) of the websites scored “somewhat easy” and 2.9% (n=2) scored “somewhat difficult” while the remaining 95.7% (n=66) scored “very difficult/no definitions found.”

Most websites show high findability for health equity/equities with 26.1% (n=18) scoring “somewhat easy” and 20.3% (n=14) scoring “somewhat difficult,” followed by health disparity/disparities with 14.5% (n=10) scoring “somewhat easy” and 17.4% (n=12) scoring “somewhat difficult.” Finally, for health inequity, 2.9% (n=2) scored “somewhat easy” and 10.1% (n=7) scored “somewhat difficult.” The remaining websites scored “very difficult/no definitions found” for health equity/equities (37/69, 53.6%), health disparity/disparities (45/69, 65.2%), and health inequity (60/69, 87%). The detailed findability scores can be found in Multimedia Appendix 2.

The overall findability results shown in Figure S2 in Multimedia Appendix 1 are a cumulative score of the findability assigned to the individual terms (see the “Methods” section). Table 1 includes the overall findability score for each website. In general, of the 69 websites we reviewed, 47.8% (n=33) scored “very difficult/no definitions found” and 36 (52.2%) scored “somewhat difficult” (Figure S2 in Multimedia Appendix 1). None of the websites had an overall score of “somewhat easy” or “very easy.” Findability results were verified by a separate researcher with a κ score of 0.77.

#### KQ2: How Are Racial Health Equity/Equities and Related Terms Included and Defined on Prominent Public Health Organization Websites?

**Figure 3.**
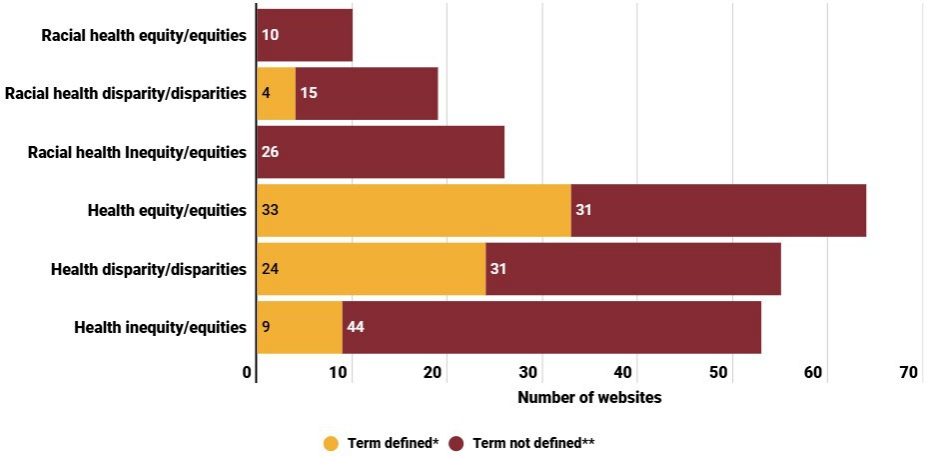
Inclusion and definition of racial health equity and related terms in reviewed websites. *Term defined indicates websites that included the terms reviewed with their definitions; **Term not defined indicates websites that included only the terms with no definitions provided.

Of the 69 websites reviewed, 15.9% (n=11) included the term “racial health equity/equities,” 37.7% (n=26) included “racial health inequity,” 29% (n=20) included “racial health disparities,” 91.3% (n=63) included “health equity/equities,” 81.2% (n=56) included “health disparity/disparities,” and 75.4% (n=52) included “health inequity.” However, only some websites contained definitions for the terms with 4.3% (n=3) defining “racial health disparity/disparities,” 47.8% (n=33) defining “health equity/equities,” 34.8% (n=24) defining “health disparity/disparities,” and 13% (n=9) defining “health inequity/inequities.” None of the websites provided definitions for the terms racial health equity/equities and racial health inequity/inequities (Figure 3). Overall, 55% (n=38) of the websites had a definition for at least 1 term; however, none had a definition for all the reviewed terms. The terms most defined together were health disparity and health equity appearing in 24.6% (n=17) of the websites (Multimedia Appendix 3).

#### KQ3: What Common Definitions, Terms, Themes, Sentiments, and Citations Related to Racial Health Equity/Equities and Related Terms Will Emerge From the Identified Health Organization Websites?

##### Thematic Analysis

**Figure 4.**
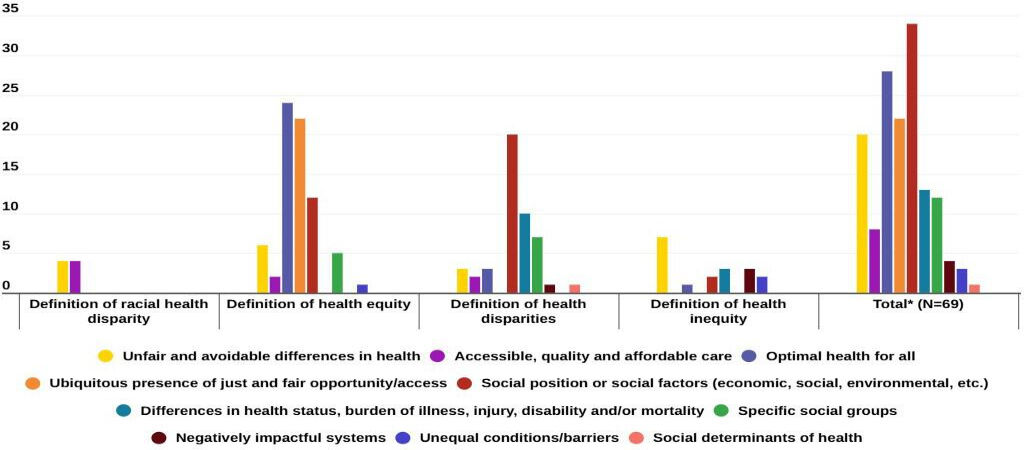
Themes identified within the definitions. *Total indicates the total number of themes from all the extracted definitions.

Overall, we found a total of 69 definitions across all websites for the included terms (racial health disparity/disparities, health equity/equities, health inequity/inequities, and health disparity/disparities). The most common theme found across all definitions was “unfair and avoidable differences” (20/69, 29%). All definitions for racial health disparity/disparities (4/4, 100%) had the themes “unfair and avoidable differences in health” and “accessible, quality, and affordable care.” Conversely, “health equity/equities” included the themes “optimal health for all” (24/33, 72.7%), “just and ubiquitous presence of fair opportunity/access” (22/33, 66.7%), and “social position or social factors (economic, social, environmental, etc)” (12/33, 36.4%) predominantly while “health disparity/disparities” had “social position or social factors (economic, social, environmental, etc)” (20/24, 88.3%), “differences in health status, burden of illness, injury, disability, and/or mortality” (10/24, 41.7%), and “specific social groups” (7/24, 29.2%) as the most common (Multimedia Appendix 4). Many of the health inequity/inequities definitions included the theme “unfair and avoidable differences in health” (7/9, 77.38). Other themes included “negatively impactful systems” (4/69, 5.8%), “unequal conditions/barriers” (3/69, 4.3%), and social determinants of health (1/69, 1.4%) (Figure 4).

##### Sentiment Analysis for Definitions

**Figure 5.**
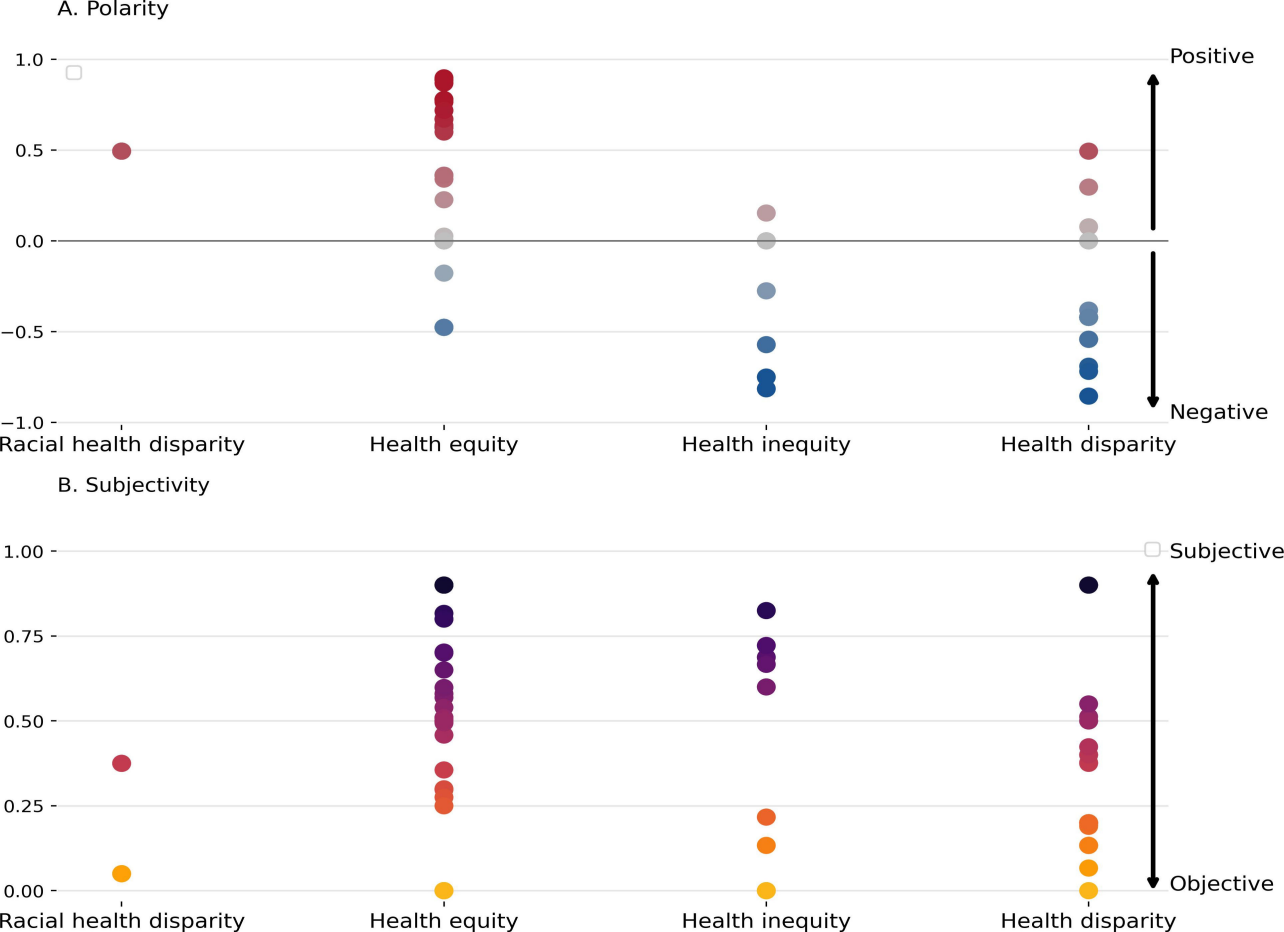
Sentiment analysis of definitions. (**A**) Polarity indicates whether the sentiment is negative (–1≤ × <–0.05), neutral (–0.05≤ × ≤0.05), or positive (0.05< × ≤1). (**B**) Subjectivity (~1) indicates that the definition is influenced by emotions while objectivity (~0) indicates no influence [51,52].

Our findings with respect to the emotional tone and subjectivity of definitions show that all the definitions for “racial health disparity/disparities” (4/4, 100%) had a positive sentiment. Likewise, most “health equity/equities” definitions (20/33, 60.6%) indicated a more positive sentiment while most definitions for “health inequity/inequities” (4/9, 44.4%) and “health disparity/disparities” (16/24, 66.7%) had a negative sentiment. Some definitions for “health equity/equities” (5/33, 15.2%), “health inequity/inequities” (4/9, 44.5%), and “health disparity/disparities” (4/24, 16.7%) had a neutral sentiment (Figure 5A). Most definitions were rated as slightly subjective (0.25‐0.75; Figure 5B). Overall, “health equity/equities” and “health inequity/inequities” definitions had average subjectivity scores of 0.5 and 0.4, respectively, while “health disparity/disparities” and “racial health disparity/disparities” definitions had average subjectivity scores of 0.3 and 0.2, respectively, making them more objective.

##### Citation Review

An additional analysis of whether the definitions were supported by citations was conducted. Of the 38 websites that contained definitions for racial health equity/equities and related terms, 44% (n=17) included 1 or more definitions that had citations. We identified 19 unique citations supporting the definitions (Multimedia Appendix 3). In total, 100% (4/4) of the racial health disparity/disparities definitions, 33.3% (11/33) of the health equity/equities definitions, 37.5% (9/24) of the health disparity/disparities definitions, and 55.6% (5/9) of the health inequity/inequities definitions included at most 1 or 2 citations. Of those definitions for racial health disparity/disparities with citations, all of them cited the “2003 Unequal Treatment report” [53]. However, the most cited works varied for other terms. The most common citations for health equity/equities were “What is Health Equity? A Definition and Discussion Guide” [54] (4/33, 12.1%), while for health disparity/disparities, 25% (6/24) cited “Healthy People 2020” [55] and 8.3% (2/24) cited “The Secretary’s Advisory Committee on National Health Promotion and Disease Prevention Objectives For 2020 Phase I Report*”* [56]. In total, 22.1% (2/9) health inequity/inequities definitions cited “The concepts and principles of equity and health” [57]. Additionally, 1 website used the same term to refer to health inequity/inequities and health disparity/disparities and cited “A glossary for health inequalities” [58]. The remaining citations [15,59-68] were cited only once per definition.

## Discussion

### Principal Results

As most Americans now seek out health information on the web [13,69], prominent health organizations have a significant role in providing credible and current evidence-based information on their public websites. In addition, definitions of racial health equity and health equity play a pivotal role in how these concepts are understood and expressed as indicated by previous studies [15].

Our study applied systematic review methods to evaluate how “racial health equity/equities” terms and definitions are presented, defined, and reported on prominent health websites. While racial health equity and related terms are frequently used on these websites, they were difficult to find and had no definitions. Those that did have definitions present varied in sentiment and underlying citations, although most of the themes were shared.

Among websites that included the reviewed terms, we determined that findability was somewhat or very difficult on all websites, indicating a lack of ease in finding the specific information sought. In addition to websites, there has been a growth of and increased access to different sources of health information, including social media, blogs, and other unverified media sources [24,27]. Additionally, the “infodemic” (too much information including false or misleading information on the web [70]) during the COVID-19 pandemic also highlighted how easily information can be misconstrued and used [70,71]. Therefore, with the various avenues available for accessing information, it is important to ensure that information on racial health equity/equities and related terms is more easily accessible to ensure common understanding and reduce ambiguity on its meaning.

The use of “racial health equity/equities” has been growing in academic literature since 2020, despite there being no clear consensus on what it means [33,35]. Definitions are particularly important for racial health equity as an emerging theoretical concept that has a significant potential for ambiguity and misunderstanding [33]. Our results showed that a definition of racial health equity/equities or racial health inequity/inequities has not yet been adopted and only 5.7% (4/69) websites defined racial health disparity/disparities. However, the results showed that there were more definitions for health equity/equities (33/69, 47.8%) and health disparity/disparities (24/69, 34.7%) while only 13% (9/69) websites had a definition for health inequity/inequities. Although these websites include health equity/equities and health disparities, there is still a gap in how these websites describe health inequities and the racialized aspect of this topic, which is increasingly important following collective calls for focused attention to health disparities in diverse racial groups, particularly after recent events (ie, COVID-19 pandemic and Black Lives Matter protests) [5,6,11].

In addition, our thematic analysis indicated that all definitions for racial health disparity/disparities (4/4, 100%), most of the health equity/equities (22/33, 66.7%), and health disparity (20/24, 83.3%) definitions share the same themes. However, most definitions were more descriptive and did not explore the causes of existing inequities. Four websites [68,72-75] contained the theme “negatively impactful systems” to access to care as the cause of inequities. Although several definitions describe “specific social groups” (12/69, 17.4%) and social factors (economic, social, and environmental factors) (34/69, 49.2%), the root causes of inequities highlighted in previous studies such as racism, historical harms, and unequal condition/barriers are not explored [53,59,76]. As these 2 concepts are intrinsically tied to addressing and achieving racial health equity/equities, including them in definitions could provide a more comprehensive understanding of the terms [59,76-78].

The sentiment analysis showed different results across websites, that is, 64.5% (20/31) of the health equity/equities definitions were positive and the remaining 35.5% (13/31) were negative or neutral. This is similarly indicated in the other definitions we found for health inequity and health disparities. A definition helps to provide a common interpretation of a concept [79]; however, inconsistency in the way definitions are perceived by the public can affect their public support and could inadvertently serve as an obstacle toward enacting policy changes [80]. Overall, definitions showed variability in content and sentiment, which could potentially lead to confusion and ambiguity among the public. Using sentiment analysis tools could be a way to initially gauge the way the information is presented and uncover potential biases.

Although few racial health disparity/disparities definitions were found, all of them cited the same source. On the other hand, most health equity/equities definitions lacked citations, although several landmark definitions have been proposed throughout the years [54,57,79]. Additionally, the definitions that contain citations use Healthy People 2020 as their primary source. However, Healthy People 2020 has been archived and replaced by the revised Healthy People 2030, which includes additional concepts such as social determinants of health [78]. Overall, the study shows that there is a lack of citations in the definitions. In addition, some of the definitions that do contain citations show promising trends by using similar references that ensure consistency across health institutes. There is, however, a need to cite up-to-date references to ensure the inclusion of new concepts that contribute to the understanding of health equity/equities and racial health equity/equities.

### Limitations

Our research was limited to websites that targeted users in the United States and that were available in English. Further research should be conducted exploring racial health equity and related terms and definitions across non-US and non–English-speaking websites. This would allow adequate comparison in understanding how racial health equity concepts are understood on a global scale. Due to the high volume of reports on the included websites, we were not able to review all reports and pages and only searched reports if results were displayed as a search result. Additionally, we only searched websites on specified dates, and since websites are being updated regularly, the searches might yield different results at different times. Additionally, since the goal of this study was to evaluate websites with a national reach, we did not evaluate state and regional health care organization websites. Consequently, sentiment analysis is limited in accurately classifying nuanced language which may lead to misclassification of sentiment.

### Conclusions

To our knowledge, this study is the first attempt to systematically evaluate racial health equity terminology and definitions in prominent health organizations’ websites. The evaluated websites are frequently used sources of health information for the public and professionals. In the websites we evaluated, we identified important gaps in incorporating and presenting racial health equity information. There was a lack of a standardized, systematic approach to presenting, defining, and using racial health equity terminology, which potentially leads to misinformation, a lack of common understanding, and contribution to the infodemic.

In the current electronic era in which the public relies on web-based information to be educated, guided, and supported in their health needs, prominent health organizations should structure their websites by following the best evidence-based information practices [29,44,69]. For that, rigorous, acceptable, and adoptable standards, guidelines, and tools are needed to assist these organizations in adequately designing, presenting, and updating information that is trustworthy and easy to find for all website consumers (eg, patients, researchers, educators, and policy makers). Therefore, having standards for prominent health organization websites for presenting definitions for health topics to be easily findable could enhance the user experience of these websites and help provide reliable information [30]. Guideline repositories, such as the Enhancing the Quality and Transparency of Health Research (EQUATOR) network, are essential for guiding unbiased and credible reporting of research results [19]; similarly, website-based networks could assist with the creation of guidelines to ensure the rigor and credibility of health information on websites. Using best evidence-based information practices (eg, use of plain language, layered information structures, and accessibility standards) [81] would increase the usability and value of health information for both the public and health care professionals, thus ensuring that maximum value is gained by ensuring adequate information about health for all. Furthermore, implementing a glossary of terms available on the home page could enhance the ease of access [82].

## Supplementary material

10.2196/64868 Multimedia Appendix 1 Inclusion and exclusion criteria and theme development samples.

10.2196/64868 Multimedia Appendix 2 Details of prominent health websites included in this study (listed alphabetically).

10.2196/64868 Multimedia Appendix 3 Definitions and citations of included terms on health websites.

10.2196/64868 Multimedia Appendix 4 Themes present with included definitions.

10.2196/64868 Checklist 1 PRISMA (Preferred Reporting Items for Systematic Reviews and Meta-Analyses) abstract checklist and PRISMA checklist for systematic review.

## Abbreviations

EQUATOR: Enhancing the Quality and Transparency of Health Research
KQ: key question
PRISMA: Preferred Reporting Items for Systematic Reviews and Meta-Analyses
