# Supplementary material for: Terminology and Definitions of Racial Health Equity in Prominent Health Websites: Systematic Review
Source: J Med Internet Res. 2025 Jul 23;27:e64868. doi: 10.2196/64868 (PMC12332040; doi:10.2196/64868)
Supplement: Multimedia Appendix 1 [file jmir-v27-e64868-s001.docx]

Figure S1. Government health organization websites traffic.


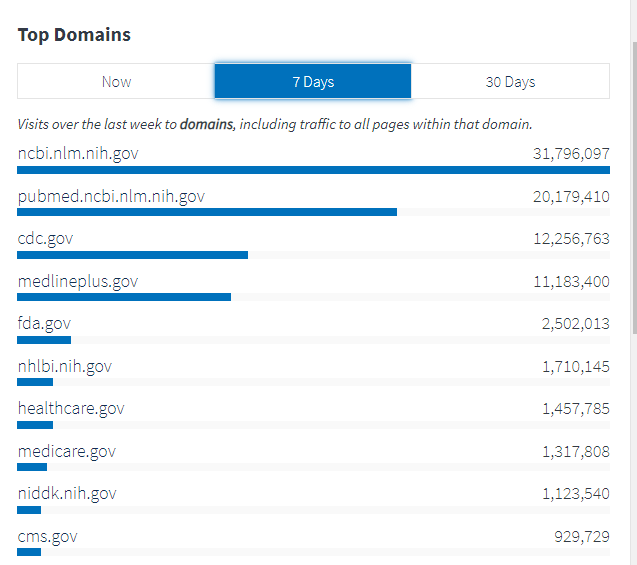


*From:* analytics.usa.gov | The US government’s web traffic. (n.d.). Analytics.Usa.Gov | The US Government’s Web Traffic. Retrieved August 24, 2023, from https://analytics.usa.gov/

Table S1. Inclusion/exclusion criteria for search strategy.

|  | **Inclusion Criteria** | **Exclusion Criteria** |
| --- | --- | --- |
| **Website type** | Credible public health organizations (ex: CDC, WHO, NIH)  Organizations that guide and inform health care (ex: CMS)  Organizations with a focus on improving health and provide best practices (ex: Cochrane)  Non-profit health organizations   - Government - National - Associations - Professional Societies | For profit health organizations (ex: pharmaceuticals)  Commercial websites  State Health Organizations  Universities  Hospitals |
| **Content** | Contains definitions of “racial health equity/ies,” “racial health inequity/ies,” or separate definitions of “health equity” within the context of race/ethnicity/minority communities | No definition of “racial health equity,” “health equity,” or related terms |
| **Setting** | Websites of health organizations in the United States or relevance to United States health care | Websites in different countries or do not guide or inform United States health care |
| **Date** | No date restriction | N/A |
| **Language** | English | Other languages |
| **Accessibility** | Publicly available to all | Website requires membership or payment to access information |

Table S2. Samples for theme development.

| **Overarching Theme** | **Code** |
| --- | --- |
| Social position or social factors (economic, social, environmental, etc.) | Social  Economic  Environmental  Socioeconomic status  Racial and ethnic background  Education level |
| Ubiquitous presence of just and fair opportunity/access | Everyone has fair and just opportunity  Everyone opportunity attain their full health potential  assurance of the conditions that allow everyone  health for all people  opportunity to be healthy  everyone has the opportunity to reach their best health. |

Figure S2. Government health organization websites' traffic.


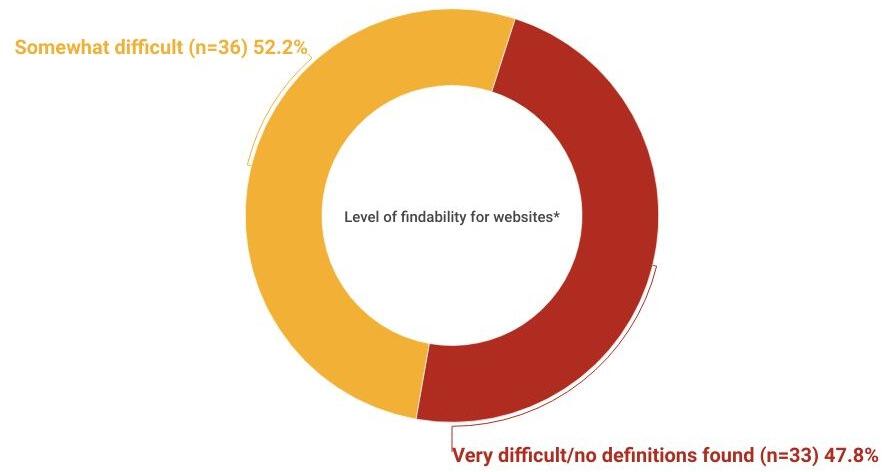


*Findability of racial health equity terms and definitions within prominent health organizations’ websites. *The level of findability for websites was assigned using the findability tool (see the “Methods” section).*
